# Combined immunodeficiency associated DOCK8 mutations

**DOI:** 10.1186/1939-4551-8-S1-A188

**Published:** 2015-04-08

**Authors:** Marcela Rezende, Laura Carneiro Matoso Nunes Canabrava, Ana Carolina Nápolis, Rodrigues Silva Segundo Segundo

**Affiliations:** 1 Universidade Federal De Uberlândia, Brazil

## Background

To present a rare case of combined immunodeficiency with high levels of IgE.

## Methods

Description of case report.

## Results

Case report: P.G.O, 9 years old, product of healthy and not consanguinity parents. At age 6 days started a recurrent intestinal bleeding, hypoactivity and fever and was hospitalized with a diagnosis of enterocolitis and sepsis by E.coli. Five days later presented cellulitis and infectious vasculitis in members with decrease of C3. Over the years presented several episodes of pneumonia, otitis, gastroenteritis, and diagnosis of atopic dermatitis. At age 3, he started skin lesions in several regions with diagnosis of poxvirus. At same time, he started warts in fingers in the hands and foot. At age 5 years, he presented atypical pneumonia and bronchiolitis obliterans with areas of atelectasis and bronchiectasis in chest tomography. Two months later, he presented malignant otitis externa and mastoiditis. Four months later, he presented abscess in the right upper limb after vacinattion to Pneumococcus. He started the follow-up in immunodeficiency out-patient clinic and the lab evaluation showed at age 4 years a normal levels of IgA,IgM and IgG, high levels of IgE (>10.000 Ui/dL), pneumococcal antibodies deficiency and low number of T cells (CD4 and CD8). The hypothesis of combined immunodeficiency associated with DOCK 8 mutations was done, because the presence of very high IgE levels, dermatological changes such as atopic dermatitis, molluscum contagiosum and warts in addiction to viral infeccions difficult control). The patient has received prophylactic cotrimoxazol since 5 years old with a significant reduction infeccions, without need new hospitalizations. He showed a normal thrive until now.

## Conclusions

It´s important to recognize the different types of primary immunodeficiency and starts the treatment to improve the quality of life and to prevent infecctions and their sequelaes.

## Consent

Written informed consent was obtained from the patient for publication of this abstract and any accompanying images. A copy of the written consent is available for review by the Editor of this journal.

